# Case of Femoral Aneurism

**Published:** 1819-02

**Authors:** Wm. Bond

**Affiliations:** Surgeon to the Norfolk and Norwich Hospital.


*For the London Medical and Physical Journal.*

*Case of Femoral Aneurism;* by WM. BOND, Esq. Surgeon to the Norfolk and Norwich Hospital.

JAMES DENNY, aged 31, of the parish of Saham Joney, Norfolk, a soldier of the fourth division of marines, five feet eight inches high, spare habit, and dark complexion, was received into the Norfolk and Norwich Hospital, June 2d, 1818, as a patient not admitting of delay, with an aneurism of the right femoral artery, the size of a large breakfast-cup, situated very high up, above Poupart's ligament: the pulsations were very evident, even to the sight, and synchronous with the heart; it gave him great pain, preventing him perfectly extending the limb. The disease is of three years' standing, and, till the last two months, never enlarged beyond the size of a nut; since which time it has rapidly increased to its present size. He can give no account of its origin; never recollects having strained himself. He has been a soldier twelve years.

On a consultation being called, on Sunday, June 6th, the operation of tying the external iliac artery was agreed upon, as being the only means of saving the life of the patient; especially as it had increased so rapidly upwards since Tuesday, the increase of two or three days more would probably have excluded the benefit of an operation. The operation was performed in the following manner, at eleven o'clock:—

The patient being placed on a table in an horizontal posture, an incision was made, five inches in length, through the integuments of the abdomen, and extending from near the anterior superior spinous process of the ilium, in an oblique direction, inwards and downwards to the bottom of the tumor; by which means the fascia of the external oblique muscle was laid bare, which, together with the internal oblique and transverse muscles, were divided by the next incision. That part of the peritoneum now exposed was divided, and the artery sought for, and easily found, and tied by a single ligature; when pulsation of the tumor instantly ceased. The outer wound was brought together by straps. When the patient first returned to his bed, (the pulse 50,) an anodyne draught was ordered. At two o'clock, pulse 54; complains of pain shooting from his hip to his knee; pulse is hurried; no sleep yet. At three o'clock, increased heat all over him; pulse 61, intermits, and is hurried; pain in the limb better. Four o'clock, pulse 61; limb, the same; he does not complain. Eight o'clock, pulse 80; heat of the operated limb 100, but two more than the sound one,—indeed, all along the heat has been pretty equal; V. S. ad. ℥xiv.: complains of pain of the thigh, and feels quite wearied; aut. rep. haust. anod.: pulse, after bleeding, 65: Twelve o'clock, pulse 70; no pain. Three o'clock, pulse 80; they have not that hurried intermitting manner: has slept pretty well, though at short intervals.

Monday morning, eight o'clock.—Passed the rest of the night pretty well; pulse 96; v. s. ad. ℥ xviij. which reduced them to 80. At eleven o'clock, pulse 100; blood much inflamed, thick buffy coat. Four o'clock, pulse 80; has had no stool since the operation; was ordered some medicine. Eight o'clock, pulse 74. Nine o'clock, pulse 100; tongue white.

Tuesday morning, eight o'clock.—Pulse 80; rep. medicam.; slept pretty well; no relief from the bowels. Eleven o'clock, pulse 80; nothing worthy of remark; both feet rather cold. Eight o'clock (evening), pulse 74; no stool yet; skin hot and dry; an enema was administered, which procured a copious evacuation, which greatly relieved him.

Wednesday morning, eight o'clock.—Slept tolerably well; complains of faintness. Eleven o'clock, pulse 80; bowels relieved; wound dressed to-day, looks healthy, discharges laudable pus. Eight o'clock (evening), pulse 74; both extremities rather cold, wrapped them up in flannel; bowels again relieved.

Thursday morning, eight o'clock.—Pulse 59; passed a bad night, was restless and uneasy; wound is painful. Eleven o'clock, wound has adhered, particularly towards the tumor, but a free opening is desirable for the exit of the discharge, which is very plentiful; pulse 80; temperature of the extremities, the same; no other alteration. Eight o'clock (evening), feels weary for want of sleep; pulse 70; great alteration in the appearance of the tumor, its volume being lessened, and softer.

Friday morning, eight o'clock.—Passed a restless night, as the bowels were not relieved yesterday; pulse 81; but the bowels were relieved this morning copiously; complains his hip is sore from lying, being obliged to lay on his right side, with the limb in the same angle, to further the discharge from the wound. Eight o'clock (evening), pulse 70; bowels again relieved this afternoon.

Saturday morning, eleven o'clock.—Pulse 76; passed a good night; complains that the wound, whilst dressing, is very painful; the temperature of the limb the same.

Sunday.—Remains the same; pulse quiet and natural; passed a good night.

Sunday, June 21st.—Every thing has gone well up to the present time: appetite good; has been kept very low; sleeps well, and the bowels regularly relieved; temperature of the limb equal; no pulsation to be felt, and no œdema. The ligature came away to-day with the dressings, being the fifteenth day since the operation. The tumor is much smaller, and altered in appearance; its feel to the touch seems broken down (if such an expression may be used), one part feeling hard and elevated, the other soft and hollow.

Tuesday, June 23d.—A surprising change has taken place in the appearance of the tumor since the last dressing, being more than one-third less, owing to a copious discharge of grumous blood from the wound, which was probably part of the contents of the aneurismal sac, finding its way upwards into the cavity of the wound; which event took place last evening, about six o'clock, accompanied by no particular pain or sensation in the tumor. The patient's apprehensions were awakened by this unexpected occurrence, which prevented him sleeping the earliest part of the night, but this morning is as well as usual; pulse 65; the angles of the wound are united, and the other parts look healthy.

Wednesday, June 24th.—The discharge of the coagulated blood is rather increased in quantity, and pressure on the tumor (which is now nearly on a level with the surrounding surface) drives the discharge through the wound, plainly showing its communication with the latter: in other respects he may be said to be well, the general system not being out of order.

Friday, June 26th.—The discharge of grumous blood is much diminished, and is also puriform; the wound is much contracted, and adhesion seems to have partly taken place in the cyst, as it is much firmer.

Monday, 29th.—The wound and integuments of the sac are very much inflamed, and have been so three days: a discutient is applied; the parts are very painful, which has prevented his sleeping for the last two nights; appetite good, pulse and secretions regular.

Tuesday, 30th.—The symptoms of suppuration of the sac are commencing, the discharge from the wound having nearly ceased, owing to the closing of that orifice from whence it issued from the sac: he was ordered opii gr. j. omni nocte.

Wednesday, July 1st.—The tumor is much enlarged to-day, from the matter collecting: it is very tender and painful in consequence; it may be expected to break in twenty-four hours; the parts are constantly wetted with the saturnine lotion.

Thursday, July 2d.—The tumor being very painful and enlarged, a common cataplasm was ordered instead of the lotion.

Friday, 3d.—The abscess burst at about twelve o'clock last night, and discharged very plentifully; the pus is of a good colour: the part is still very painful.

Monday, 6th.—The patient sits up to-day for the first time: the abscess has nearly done discharging; the granulations of the wound are above the surface, and require to be repressed by escharotics.

Friday, 10.—He was allowed meat and beer. The tumor has continued to decrease, and he is going on well.

From this time until the 8th of August, he continued gradually to regain his health. He walks without any difficulty, and was this day discharged cured.

*Norwich; Nov.* 16, 1818.

